# An Optical Coherence Tomography-Based Deep Learning Algorithm for Visual Acuity Prediction of Highly Myopic Eyes After Cataract Surgery

**DOI:** 10.3389/fcell.2021.652848

**Published:** 2021-05-26

**Authors:** Ling Wei, Wenwen He, Jinrui Wang, Keke Zhang, Yu Du, Jiao Qi, Jiaqi Meng, Xiaodi Qiu, Lei Cai, Qi Fan, Zhennan Zhao, Yating Tang, Shuang Ni, Haike Guo, Yunxiao Song, Xixi He, Dayong Ding, Yi Lu, Xiangjia Zhu

**Affiliations:** ^1^Department of Ophthalmology, Eye and ENT Hospital, Eye Institute, Fudan University, Shanghai, China; ^2^Key Laboratory of Myopia, NHC Key Laboratory of Myopia, Fudan University, Chinese Academy of Medical Sciences, Shanghai, China; ^3^Shanghai Key Laboratory of Visual Impairment and Restoration, Shanghai, China; ^4^Visionary Intelligence Ltd, Beijing, China; ^5^Department of Ophthalmology, Heping Eye Hospital, Shanghai, China; ^6^Illinois Computer Science, University of Illinois, Champaign, IL, United States

**Keywords:** machine learning, visual acuity, high myopia, cataract, optical coherence tomography

## Abstract

**Background:**

Due to complicated and variable fundus status of highly myopic eyes, their visual benefit from cataract surgery remains hard to be determined preoperatively. We therefore aimed to develop an optical coherence tomography (OCT)-based deep learning algorithms to predict the postoperative visual acuity of highly myopic eyes after cataract surgery.

**Materials and Methods:**

The internal dataset consisted of 1,415 highly myopic eyes having cataract surgeries in our hospital. Another external dataset consisted of 161 highly myopic eyes from Heping Eye Hospital. Preoperative macular OCT images were set as the only feature. The best corrected visual acuity (BCVA) at 4 weeks after surgery was set as the ground truth. Five different deep learning algorithms, namely ResNet-18, ResNet-34, ResNet-50, ResNet-101, and Inception-v3, were used to develop the model aiming at predicting the postoperative BCVA, and an ensemble learning was further developed. The model was further evaluated in the internal and external test datasets.

**Results:**

The ensemble learning showed the lowest mean absolute error (MAE) of 0.1566 logMAR and the lowest root mean square error (RMSE) of 0.2433 logMAR in the validation dataset. Promising outcomes in the internal and external test datasets were revealed with MAEs of 0.1524 and 0.1602 logMAR and RMSEs of 0.2612 and 0.2020 logMAR, respectively. Considerable sensitivity and precision were achieved in the BCVA < 0.30 logMAR group, with 90.32 and 75.34% in the internal test dataset and 81.75 and 89.60% in the external test dataset, respectively. The percentages of the prediction errors within ± 0.30 logMAR were 89.01% in the internal and 88.82% in the external test dataset.

**Conclusion:**

Promising prediction outcomes of postoperative BCVA were achieved by the novel OCT-trained deep learning model, which will be helpful for the surgical planning of highly myopic cataract patients.

## Introduction

A predicted number of 938 million people of the world’s population may suffer from high myopia by the year 2050 (Holden et al., 2016), leading to a major worldwide concern. Eyes with high myopia were prone to early-onset and nuclear-type cataracts (Hoffer, 1980; Zhu et al., 2018). Yet, nowadays, surgery is the only effective therapeutic method for cataracts (Thompson and Lakhani, 2015). With the advancement of techniques, cataract surgery can now provide a promising visual outcome in nonmyopes (Liu et al., 2017). However, for highly myopic cataract patients, due to the more complicated fundus conditions such as foveoschisis, chorioretinal atrophy, or cicatrices from previous choroidal neovascularization (Chang et al., 2013; Todorich et al., 2013; Gohil et al., 2015; Lichtwitz et al., 2016; Li et al., 2018), their visual benefit from cataract surgery remains hard to be determined preoperatively.

With the wide application of optical coherence tomography (OCT), surgeons could assess the fundus status of highly myopic eyes on an anatomical scale (Huang et al., 2018; Li et al., 2018), but the morphological diagnoses were hard to be directly associated with the actual manifested visual acuity (VA). Therefore, difficulties might occur when surgeons want to predict the postoperative VA and explain the prognosis to the highly myopic patients during preoperative conversations, which might thereby affect the overall surgical planning and patients’ satisfaction with the surgery later.

Recently, deep learning was found promising in automated classification. Particularly, the ResNet and Inception algorithms have their advantages on medical image analysis (Gulshan et al., 2016; Fu et al., 2018; Grassmann et al., 2018). Such techniques have the potential to revolutionize the diagnosis and clinical prediction by rapidly reviewing large amounts of morphological features and by performing integrations difficult for human experts (Kermany et al., 2018). Hence, prediction of the clinical manifestation based on deep learning analysis of relevant morphological features is becoming possible and important (Chen et al., 2018; Rohm et al., 2018). However, due to the more complicated and variable morphological changes of the fundus, no appropriate deep learning model has been developed for highly myopic eyes currently.

In this study, on the basis of the OCT scans of highly myopic eyes, we aim to predict their postoperative VA of cataract surgery by developing and comparing five machine learning algorithms and consequently evaluating the model on real-world datasets.

## Materials and Methods

### Ethics

The Institutional Review Board of the Eye and Ear, Nose, and Throat (ENT) Hospital of Fudan University (Shanghai, China) approved this study. The study adhered to the tenets of the Declaration of Helsinki and was registered at www.clinicaltrials.gov (accession number NCT03062085). Written consent was obtained from the patients and all private information was removed in advance.

### Patients

An internal dataset including 1,415 highly myopic eyes from 1,415 patients was drawn from the database of the Shanghai High Myopia Study between 2015 and 2020 at the Eye and ENT Hospital of Fudan University (Shanghai, China). Eligible criteria were as follows: (1) cataract patients with axial length (AL) over 26.0 mm, (2) had reliable macular OCT measurements before cataract surgery, (3) underwent uneventful cataract surgeries, and (4) had credible postoperative best corrected visual acuity (BCVA) measured at 4 weeks after surgery. Exclusion criteria were eyes with (1) corneal opacity or other corneal diseases that may significantly influence the visual pathway, (2) congenital ocular abnormities, (3) neuropathies that may influence the visual acuity, (4) ocular trauma, and (5) other severe oculopathies that may affect the surgical outcomes. The OCT images in the internal dataset were taken from Spectrialis OCT (Heidelberg Engineering, Heidelberg, Germany) or Cirrus OCT (Carl Zeiss Meditec, Dublin, CA, United States).

Another external dataset consisted of 161 highly myopic eyes of 161 patients drawn from the database of the Heping Eye Hospital (Shanghai, China) with the same inclusion and exclusion criteria. The OCT images in this external dataset were taken from Spectrialis OCT (Heidelberg Engineering, Heidelberg, Germany).

### Datasets

The eligible internal database was randomly divided into a training dataset, a validation dataset, and an internal test dataset with a fixed ratio of 6:2:2. The eligible external database was all used as an external test dataset. The actual BCVAs at 4 weeks after cataract surgery were set as the ground truth. The Snellen VA was converted to its logarithm of minimal angle of resolution (logMAR) equivalent as previously described, with counting fingers being assigned a value of 1.9, hand motion 2.3, light perception 2.7, and no light perception 3.0 (Lange et al., 2009). Eyes with actual BCVAs < 0.30 logMAR (Snellen 6/12 or higher) were defined as the good VA group, while eyes with actual BCVAs ≥ 0.30 logMAR (Snellen 6/12 and lower) were defined as the poor VA group (Quek et al., 2011).

### Data Normalization

The e2e files from Spectrialis OCT or scan figures from Cirrus OCT were extracted and preprocessed. All OCT images were down-sized to 224 × 224 pixels, the default choice for deep learning-based image classification. In order to simulate more real-world situations and to improve model generalization ability, it was performed on the image by changing the brightness, saturation, and contrast with a factor uniformly sampled from [0, 2], respectively. After the color space normalization, the macular OCT images were set as model input.

### Deep Learning Models

In our study, to predict the BCVA after cataract surgery for highly myopic patients, we constructed an ensemble learning using five different deep convolutional neural networks (CNN) algorithms, including Deep Residual Learning for Image Recognition (ResNet, Microsoft Research) with 18, 34, 50, and 101 layers (ResNet-18, ResNet-34, ResNet-50, and ResNet-101) (He et al., 2016) and Inception-v3 (Szegedy et al., 2016). The postfix number of ResNet referred to diverse depths of ResNet networks that lead to different parameter scales. All five models were pretrained on the ImageNet dataset. For each model, the last fully connected layer which originally output 1,000 class was replaced to output a single value to suit our task. The parameters of this layer were randomly initialized.

Based on the training dataset, the model was optimized with a target of minimizing the mean square error (MSE) loss function using the Adam optimizer (Fu et al., 2018). The final output score was calculated as the mean value of the ensemble model. MSE loss was defined as:

(1) $$M\,S\,E=\frac{1}{N}\,\sum_{i=1}^{N}{\left(\tilde{y_{i}}-y_{i}\right)}^{2},$$

where *N* indicates the number of input OCT images, $\tilde{y_{i}}$ indicates the actual BCVA, and *y*_*i*_ indicates the predicted BCVA.

The maximal number of training epochs was set to be 80. We adopted an early stop strategy, which is the training procedure stops when there is no performance improvement on the validation dataset in 15 consecutive epochs. The initial learning rate was set to 0.001 and would be decayed by 0.1 every 30 epochs. Each CNN algorithm was trained five times repeatedly, and only the model with the best performance on the validation dataset was reserved for the ensemble learning.

### Evaluation

The metrics used to show the differences in logMAR postoperative BCVA between the prediction and the ground truth were mean absolute error (MAE, calculated for the predictions of the algorithms compared to the ground truth) and the root mean square error (RMSE), which were defined as:

(2) $$\text{MAE}=\frac{1}{N}\,\sum_{i=1}^{N}\left|\tilde{y_{i}}-y_{i}\right|,$$

(3) $$\text{RMSE}=\sqrt{\frac{1}{N}\,\sum_{i=1}^{N}{\left(\tilde{y_{i}}-y_{i}\right)}^{2}},$$

where *N*, $\tilde{y_{i}}$, and *y_i_* were defined as above.

Furthermore, sensitivity is defined as the proportion of correctly predicted eyes with VA < 0.30 logMAR (or ≥0.30 logMAR) in the overall eyes having actual VA < 0.30 logMAR (or ≥0.30 logMAR). Precision is defined as the proportion of correctly predicted eyes with VA < 0.30 logMAR (or ≥0.30 logMAR) in the overall eyes having predicted VA < 0.30 logMAR (or ≥0.30 logMAR).

The ensemble learning of the five CNN models was adopted to develop the prediction model and then further evaluated using the internal and external test datasets, which contain data the model has not seen. The OCT reports in pdf format from both test datasets were adopted and evaluated. The prediction error was calculated by subtracting the predicted BCVA from the actual BCVA. The percentage of BCVA prediction errors within ± 0.30 logMAR (Snellen 6/12, *R*_e0.30 logMAR_) was then calculated (Gao et al., 2015), which was defined as:

(4) R=e0⁢.3⁢logMAR1N∑i=1NI(|yi~-yi|≤0.3logMAR),

where *N*, $\tilde{y_{i}}$, and *y_i_* were defined as above. *I*(⋅) is the function which returns 1 if the ⋅ is true, else return 0.

In order to make the performance more comparable, fixed randomly generated seeds were used to shuffle the data and initialize the models’ parameter. To better visualize the prediction, gradient-weighted class activation mapping (Grad-CAM) was used to highlight the model’s interests in the OCT images in prediction VA (Selvaraju et al., 2020).

**FIGURE 1:**
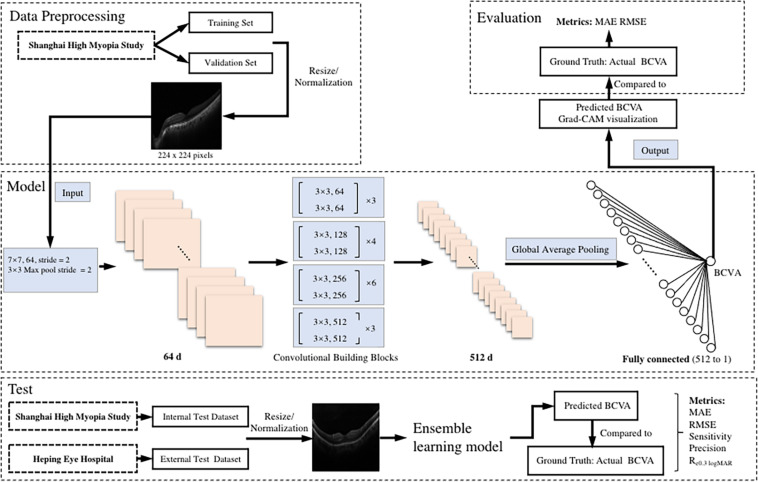
An illustration of the pipeline of the tasks. The preoperative b-scan OCT image is fed into the model. It eventually outputs the prediction of postoperative BCVA. BCVA, best corrected visual acuity; logMAR, logarithm of the minimum angle of resolution; MAE, mean absolute error; RMSE, root mean square error; R_e0.30 logMAR_, the percentage of BCVA prediction errors within ± 0.30 logMAR.

The illustration of the pipeline of our work is demonstrated in Figure 1.

### Statistics

Continuous variables were described as the mean ± standard deviation. The Student’s *t* test or one-way ANOVA test followed by Tukey’s test was used to compare the continuous variables and the χ^2^ test was used to compare categorical variables. The alignment of the predicted BCVA and ground truth was demonstrated by scatter plots. Pearson correlation analysis was used to evaluate the relationship between the predicted outcome and the ground truth, and the Bland–Altman plot was used to assess the agreement between the predicted outcome and the ground truth. The information of the computer used in this study was as follows: Intel Xeon 4144 (2.20 GHz), 128 gigabytes of RAM, and three pieces of GeForce RTX 2080 Ti Ubuntu 18.04 LTS. Model development was performed by Python (version 3.7.5) with libraries of torch (version 1.4.0) and torchvision (version 0.4.0), and statistical analyses were performed with a commercially available statistical software package (SPSS Statistics 20.0; IBM, Armonk, NY).

## Results

The clinical characteristics of the patients are demonstrated in Table 1. No difference was found in age, sex, and mean actual postoperative BCVA among the training, validation, internal test, and external test datasets (*p* > 0.05).

**TABLE 1:** Demographic and clinical characteristics.

	Internal datasets			External test dataset
	
	Training	Validation	Test	
Number of eyes	851	282	282	161
Female gender (%)	391 (45.9%)	158 (56.0%)	150 (53.2%)	86 (53.4%)
Age (mean ± SD, years)	61.37 ± 10.45	61.93 ± 11.15	61.19 ± 9.47	62.45 ± 9.32
Actual postoperative BCVA (LogMAR, mean ± SD)	0.26 ± 0.33	0.25 ± 0.30	0.25 ± 0.31	0.14 ± 0.19
**Number of OCT images in each BCVA range**				
<0.30 logMAR (Snellen 6/12 or higher)	559	186	186	137
≥0.30 logMAR (Snellen 6/12 or lower)	292	96	96	24

*LogMAR, logarithm of the minimum angle of resolution; BCVA, best corrected distance visual acuity.*

The performances of all five CNN algorithms were compared after training and validating for five times. The average values of the five-time performances of the five models separately and the ensemble learning outcomes combining all models’ decisions using the validation dataset are presented in Table 2. Notably, the ensemble learning showed the lowest MAE (0.1566 logMAR) and the lowest RMSE (0.2433 logMAR). Therefore, the ensemble learning model with the most promising performance was then chosen for further development and evaluations.

**TABLE 2:** The performances of five algorithms and the ensemble learning using the validation dataset (*n* = 282).

Algorithms	ResNet-18	ResNet-34	ResNet-50	ResNet-101	Inception-v3	Ensemble
MAE	0.1648	0.1737	0.1729	0.1723	0.1842	0.1566*
RMSE	0.2540	0.2677	0.2682	0.2600	0.2857	0.2433*

MAE, mean absolute error; RMSE, root mean square error.

*The best performances in MAE and RMSE.

**FIGURE 2:**
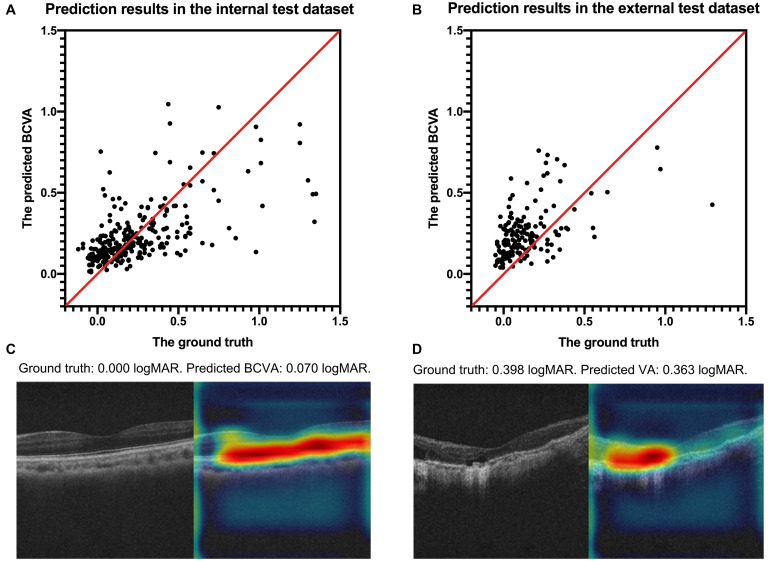
The scatter plots of the predicted BCVA and the actual BCVA (ground truth) in the internal **(A)** and external **(B)** test datasets. Representative cases of Grad-CAM visualization in the good VA group **(C)** and in the poor VA group **(D)**. Red regions corresponds to highly discriminative areas of OCT scans when predicting the VA. All values were provided in logMAR units. BCVA, best corrected distance visual acuity; logMAR, logarithm of the minimum angle of resolution; Grad-CAM, gradient-weighted class activation mapping.

The internal and external test datasets were used to determine the performance of our prediction model using the ensemble learning and to confirm the generalizability. As shown in Table 3, the prediction model demonstrated stably promising outcomes with MAEs of 0.1524 and 0.1602 logMAR and RMSEs of 0.2612 and 0.2020 logMAR in the internal and external test datasets, respectively. In the internal test dataset, the sensitivity of our model was 90.32% in the good VA group and 42.71% in the poor VA group; the precision was 75.34% in the good VA group and 69.49% in the poor VA group. In the external test dataset, the sensitivity of our model was 81.75% in the good VA group and 45.83% in the poor VA group; the precision was 89.60% in the good VA group and 30.55% in the poor VA group. The scatter plot of the predicted BCVA and the ground truth (actual BCVA) was demonstrated in the internal (Figure 2A) and external test datasets (Figure 2B). Pearson correlation analysis revealed the significant relationships between the predicted BCVA and the ground truth in the internal test dataset (*r* = 0.55; *p* < 0.001) and external test dataset (Pearson coefficients *r* = 0.50; *p* < 0.001). The Grad-CAM visualization was used for the CNN models. Representative cases in the good VA group (Figure 2C) and in the poor VA group (Figure 2D) were demonstrated, showing the highly discriminative region of OCT scans when predicting the VA.

**TABLE 3:** The performances of the prediction model in the internal (*n* = 282) and external test datasets (*n* = 161).

Algorithms	Internal test dataset	External test dataset
MAE	0.1524	0.1602
RMSE	0.2612	0.2020
Sensitivity in each VA group*		
<0.30 logMAR (Snellen 6/12 or higher)	90.32% (168/186)	81.75% (112/137)
≥0.30 logMAR (Snellen 6/12 and lower)	42.71% (41/96)	45.83% (11/24)
**Precision in each VA group^†^**		
<0.30 logMAR (Snellen 6/12 or higher)	75.34% (168/223)	89.60% (112/125)
≥0.30 logMAR (Snellen 6/12 and lower)	69.49% (41/59)	30.55% (11/36)

MAE, mean absolute error; RMSE, root mean square error.

*Sensitivity = number of correctly predicted eyes with VA < 0.30 logMAR (or ≥0.30 logMAR)/overall number of eyes having actual VA < 0.30 logMAR (or ≥0.30 logMAR).

*^†^Precision = number of correctly predicted eyes with VA < 0.30 logMAR (or ≥0.30 logMAR)/overall number of eyes having predicted VA < 0.30 logMAR (or ≥0.30 logMAR).*

**FIGURE 3:**
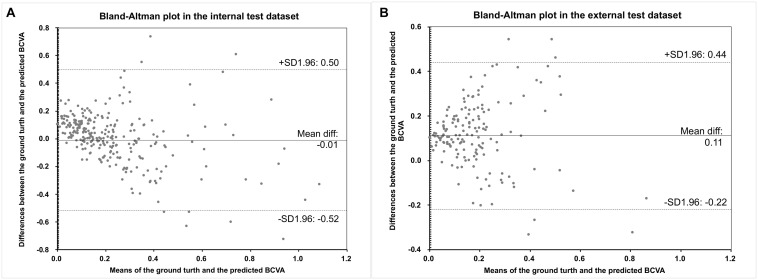
The Bland–Altman plots of the predicted BCVA and the actual BCVA (ground truth) in the internal **(A)** and external **(B)** test datasets. All values were provided in logMAR units. BCVA, best corrected distance visual acuity; logMAR, logarithm of the minimum angle of resolution.

**FIGURE 4:**
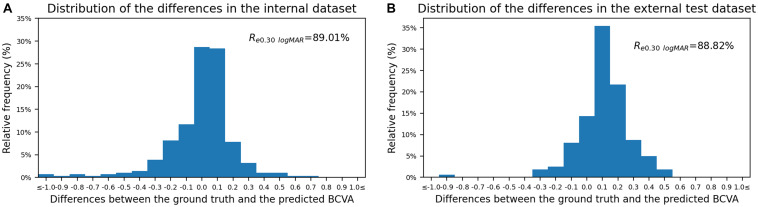
The distribution of the difference between the predicted BCVA and the actual BCVA (ground truth) in the internal **(A)** and external **(B)** test datasets. All values were provided in logMAR units. The vertical axis indicates the relative frequency of each BCVA delta value. BCVA, best corrected visual acuity; logMAR, logarithm of the minimum angle of resolution; *R*_*e0.30 logMAR*_, the percentage of BCVA prediction errors within ± 0.30 logMAR.

The Bland–Altman plots assessing the agreement between predictions and the ground truth are shown in Figure 3. The 95% confidence limits of agreement ranged from −0.52 to 0.50 logMAR in the internal test dataset and −0.22 to 0.44 logMAR in the external test dataset, while no statistically significant evidence of proportional bias was found (both *p* > 0.05). Figure 4 shows the distributions of the difference between the ground truth and the predicted BCVA in both test datasets. The percentages of the prediction errors within ± 0.30 logMAR were 89.01% in the internal test dataset and 88.82% in the external test dataset.

Further analysis was conducted on the falsely predicted cases in the test datasets. They can be divided into two groups: (1) underestimated cases: the ground truth < 0.30 logMAR (good VA) but the predicted VA ≥ 0.30 logMAR (poor VA), and (2) overestimated cases: the ground truth ≥ 0.30 logMAR (poor VA) but the predicted VA < 0.30 logMAR (good VA). Supplementary Table 1 shows the distribution of all falsely predicted cases. These cases can be attributed to the following four categories: (A) vague OCT images induced by extraordinarily cloudy cataract (under- or overestimated, 39.6%); (B) morphological changes on OCT scan exist but might have poor effect on VA (underestimated, 8.1%), e.g., changes located away from the macular, which were irregularly focused by the model; (C) morphological changes on OCT scan exist but might have unclear effect on VA (under- or overestimated VA, 46.8%), e.g., rough retinal pigment epithelium layer or irregular inner segment/outer segment layer; and (D) morphological changes on OCT scan exist which might have some effect on VA, but were presented as signal-deficient lesions and were accidentally ignored by the model (overestimated VA, 5.4%). Representative cases in the four categories with their Grad-CAM visualizations are presented in Supplementary Figure 1.

## Discussion

Highly myopic cataract patients usually inevitably have macular complications such as foveoschisis, chorioretinal atrophy, and cicatrices from previous choroidal neovascularization (Chang et al., 2013; Todorich et al., 2013; Gohil et al., 2015; Lichtwitz et al., 2016; Li et al., 2018), which could render the preoperative prediction of visual acuity after cataract surgery very difficult, even though an OCT scan can be used for morphological diagnosis (Jeon and Kim, 2011). In the present study, by using the preoperative OCT scans of macular as input, we developed and validated a deep learning algorithm to predict the postoperative BCVA of highly myopic eyes after cataract surgery and revealed that the ensemble model showed stably promising performance in both internal and external test datasets with MAEs of 0.1524 and 0.1602 logMAR and RMSEs of 0.2612 and 0.2020 logMAR, respectively.

Cataract patients usually expect a significant improvement of VA after removal of the cloudy lens (Zhu et al., 2017). However, those with high myopia are more concerned about their VA improvement during the surgical planning stage. Myopic maculopathies are the main source of the gap between the expected outcomes and the actual potentials their fundus have. Hence, a forecast model which could tell the patients their potential postoperative visual acuities might be helpful with their surgical decisions (Rönbeck et al., 2011). Nevertheless, the prediction of VA for highly myopic eyes has always been very difficult. Although high-resolution OCT may reveal morphological changes and thereby identify eyes at high risk of developing clinically significant macular complications affecting the postoperative visual outcomes (Hayashi et al., 2010), it is still hard for cataract surgeons to specifically determine the exact postoperative VA preoperatively.

In recent years, deep learning has been widely applied for its ability to process highly complex tasks through a neural network, which can be seen as a mathematical function composed of a large number of parameters provided by medical images. An OCT scan of macular could provide millions of morphological parameters affecting the VA (Abdolrahimzadeh et al., 2017; Chung et al., 2019). The neural network was able to identify the corresponding features and thereby automatically generate the target VA predictions. Therefore, using deep learning algorithms to predict the postoperative BCVA was practicable and meaningful. Compared with other types of neural networks, CNN can initially identify a few adjacent pixels as local lower-level features and then merge them into global higher-level features, and thus, it has been proven effective widely in the field of medical image analysis (Anwar et al., 2018). In the current study, when taking a preoperative OCT image as input, the ensemble learning showed the most promising performance, and the model automatically predicted the postoperative BCVA for highly myopic eyes having cataract surgeries with promising accuracies. With this model, surgeons only need to input an OCT image of macular, and a predicted postoperative BCVA together with a Grad-CAM visualization could be generated. The expectant surgical outcomes could be discussed between the surgeons and highly myopic patients before surgery. Patients might more thoroughly understand their macular status and how it might affect the visual outcome of cataract surgery. It might also help with surgical decisions such as whether to choose premium IOL implantations.

Previous reports about applying deep learning approaches to predict VA outcomes were mainly in the field of retinal or macular diseases, such as age-related macular degeneration, diabetic retinopathy, or retinopathy of prematurity (Chen et al., 2018; Rohm et al., 2018; Huang et al., 2020). The morphologies of featured lesions for these diseases were relatively simple or identifiable. Yet, there are rare studies about implementing the deep learning approach on high myopia due to its more complicated and variable fundus status (Zhu et al., 2020). It might be more valuable to predict the VA outcomes based on the diverse fundus morphologies for highly myopic eyes. Moreover, dozens of features from the patients’ medical history were adopted or annotated one by one to train their models (Chen et al., 2018; Rohm et al., 2018; Huang et al., 2020). It might be highly difficult to ensure that during applications, such many features from real-world patients were available simultaneously and completely. The data missing problem might be serious and may result in uncertain accuracies, thus restricting the generalizability of their models. Our study, mainly targeting VA prediction of highly myopic eyes, adopted the OCT scan as the only input feature, which examines almost every highly myopic patient before their cataract surgery. Hence, the data missing problem could be rare when clinically applying our model.

Notably, our model has shown considerable sensitivity and precision in the good VA group in both test datasets (all >75%), thus solving nearly 60% of the problems after cataract surgery according to a previous report (Barañano et al., 2008). As for the poor VA group, the model demonstrated relatively lower sensitivity and precision, which might due to the very complicated and changeable characteristic of the fundus status among these highly myopic patients. As for the falsely predicted cases in categories A and C, manually predicting the VA can still be tricky for experienced cataract surgeons. In the future, the accuracy could be further improved by the model training with larger sample sizes. As for the falsely predicted cases in categories B and D, they revealed less focus on the signal-deficient signs by the model intrinsically, but only made up very minimal proportions. This can be further improved by manual annotations of the signal-deficient lesions when more cases are included in model training in the future. Currently, as there are no perfect ways to accurately predict the surgical benefit of highly myopic patients with very poor fundus condition, our predictions by the deep learning model might still provide valuable references for preoperative communications and clinical decisions for this special population.

In conclusion, based on macular OCT images taken before cataract surgery, we are taking the lead to originally develop the deep learning prediction model for highly myopic eyes, which can provide promising predictions of postoperative BCVA for cataract patients with high myopia. Our model will be helpful for surgical planning and preoperative conversations with highly myopic cataract patients.

## Data Availability Statement

The raw data supporting the conclusions of this article will be made available by the authors, without undue reservation.

## Ethics Statement

The studies involving human participants were reviewed and approved by The Institutional Review Board of the Eye and Ear, Nose, and Throat Hospital of Fudan University (Shanghai, China). The patients/participants provided their written informed consent to participate in this study.

## Author Contributions

LW and WH collected the data, performed the analyses, and wrote the manuscript. JW, XH, and DD programmed the model and performed the analyses. KZ, YD, JQ, and JM collected the data and performed the analyses. XQ, LC, QF, ZZ, YT, SN, and HG collected the data. YS performed the analyses. YL and XZ gained the fund and supervised the process. XZ revised the manuscript, gained the fund, and supervised the process. All authors contributed to the article and approved the submitted version.

## Conflict of Interest

JW, XH, and DD were employed by the company Visionary Intelligence Ltd. The remaining authors declare that the research was conducted in the absence of any commercial or financial relationships that could be construed as a potential conflict of interest.
