# Supplementary material for: An Optical Coherence Tomography-Based Deep Learning Algorithm for Visual Acuity Prediction of Highly Myopic Eyes After Cataract Surgery
Source: Front Cell Dev Biol. 2021 May 26;9:652848. doi: 10.3389/fcell.2021.652848 (PMC8187805; doi:10.3389/fcell.2021.652848)
Supplement: Supplementary file 1 [file Data_Sheet_1.PDF]

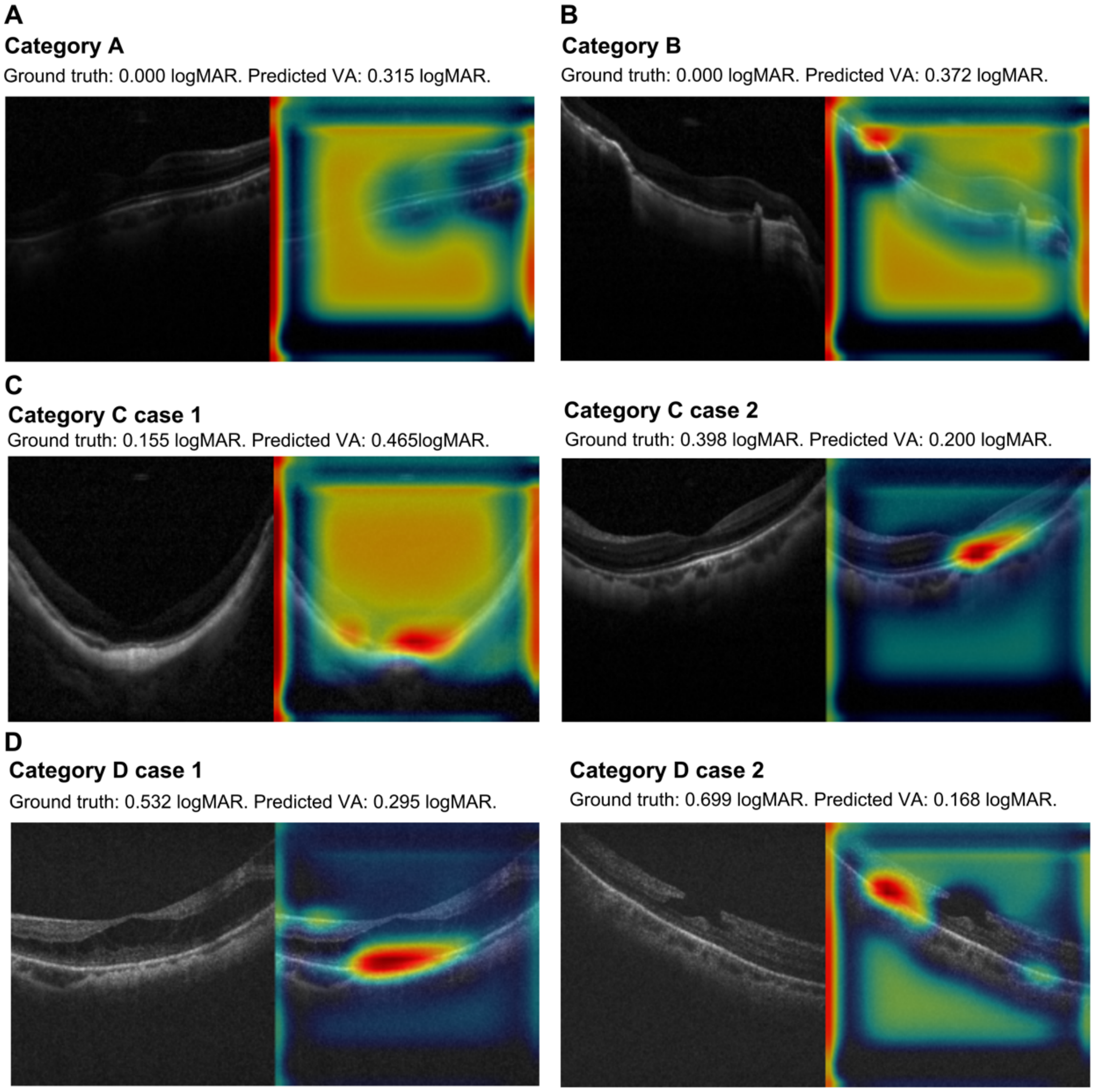

Supplemental Figure 1. The representative falsely-predicted cases and Grad-CAM visualizations in 4 categories. **A.** A case in Category A shows a vague OCT image induced by extraordinarily cloudy cataract. **B**. A case in Category B shows a morphological change on OCT scan but located away from the macular, which might have poor effect on VA, and was irregularly focused by the model. **C.** Two cases in Category C shows morphological changes on OCT scan but might have unclear effect on VA. Case 1 shows a rough retinal pigment epithelium layer, while case 2 shows an irregular inner segment/outer segment layer. **D**. Two Cases in Category D shows signal-deficient changes on OCT scan which might have some effect on VA, but accidentally ignored by the model. Case 1 shows a cystoid macular edema, while case 2 shows a macular hole. Red regions corresponds to highly discriminative areas of OCT scans when predicting the VA. All values were provided in logMAR units. BCVA= best corrected distance visual acuity; logMAR = logarithm of the minimum angle of resolution; Grad-CAM = Gradient-weighted Class Activation Mapping.
