# Supplementary material for: An Optical Coherence Tomography-Based Deep Learning Algorithm for Visual Acuity Prediction of Highly Myopic Eyes After Cataract Surgery
Source: Front Cell Dev Biol. 2021 May 26;9:652848. doi: 10.3389/fcell.2021.652848 (PMC8187805; doi:10.3389/fcell.2021.652848)
Supplement: Supplementary file 2 [file Table_1.DOCX]

| Number of falsely-predicted cases | Internal | External | Total | Categories* | | | |
| --- | --- | --- | --- | --- | --- | --- | --- |
|  |  |  |  | A | B | C | D |
| Underestimated | 18 | 25 | 43 | 15 (34.9%) | 9 (20.9%) | 19 (44.2%) | - |
| Overestimated | 55 | 13 | 68 | 29 (42.6%) | - | 33 (48.5%) | 6 (8.8%) |
| Total | 73 | 38 | 111 | 44 (39.6%) | 9 (8.1%) | 52 (46.8%) | 6 (5.4%) |

**Supplemental Table 1.** Numbers and percentages of falsely-predicted cases in under- and overestimated groups.

*Categories were divided as:

A) vague OCT images induced by extraordinarily cloudy cataract;

B) morphological changes on OCT scan exists but might have poor effect on VA;

C) morphological changes on OCT scan exists but might have unclear effect on VA;

D) morphological changes on OCT scan exists which might have some effect on VA, but were presented as signal-deficient lesions and were accidentally ignored by the model.
